# Within-Day Amino Acid Intakes and Nitrogen Balance in Male Collegiate Swimmers during the General Preparation Phase

**DOI:** 10.3390/nu10111809

**Published:** 2018-11-20

**Authors:** Takeshi Matsuda, Hiroyuki Kato, Haruka Suzuki, Ami Mizugaki, Takahiko Ezaki, Futoshi Ogita

**Affiliations:** 1 Department of Sports and Life Sciences, National Institute of Fitness and Sports, 1 Shiromizu-cho, Kanoya, Kagoshima 891-2393, Japan; matsutake0623@aol.com; 2 Frontier Research Laboratories, Institute for Innovation, Ajinomoto Co., Inc., 1-1 Suzuki-cho, Kawasaki, Kanagawa 210-8681, Japan; hiroyuki_kato@ajinomoto.com (H.K.); ami_mizugaki@ajinomoto.com (A.M.); 3 Olympic and Paralympic Promotional Office, Ajinomoto Co., Inc., 1-15-1 Kyobashi, Tokyo 210-8681, Japan; haruka_suzuki@ajinomoto.com (H.S.); takahiko_ezaki@ajinomoto.com (T.E.)

**Keywords:** protein requirement, nitrogen balance, leucine, swimming

## Abstract

A higher protein intake is recommended for athletes compared to healthy non-exercising individuals. Additionally, the distribution and quality (i.e., leucine content) of the proteins consumed throughout the day should be optimized. This study aimed to determine the nitrogen balance and distribution of protein and amino acid intakes in competitive swimmers during the general preparation phase. Thirteen swimmers (age: 19.7 ± 1.0 years; VO_2_max: 63.9 ± 3.7 mL·kg^−1^·min^−1^, mean ± standard deviation) participated in a five-day experimental training period. Nutrient intakes were assessed using dietary records. Nitrogen balance was calculated from the daily protein intake and urinary nitrogen excretion. The intake amounts of amino acids and protein at seven eating occasions were determined. The average and population-safe intakes for zero nitrogen balance were estimated at 1.43 and 1.92 g·kg^−1^·day^−1^, respectively. The intake amounts of protein and leucine at breakfast, lunch, and dinner satisfied current guidelines for the maximization of muscle protein synthesis, but not in the other four occasions. The population-safe protein intake level in competitive swimmers was in the upper range (i.e., 1.2–2.0 g·kg^−1^·day^−1^) of the current recommendations for athletes. The protein intake distribution and quality throughout the day may be suboptimal for the maximization of the skeletal muscle adaptive response to training.

## 1. Introduction

Swimming is a sport demanding muscle strength and endurance capacity underpinned by different combinations of anaerobic and aerobic fuel systems. To improve their endurance capacity, competitive swimmers generally participate in a large volume of endurance training in combination with low-intensity and high-intensity exercise [1]. Competitive swimmers train for 3–4 h·day^−1^ and swim up to 10,000 m·day^−1^ [2]; in particular, world-class swimmers swim up to 17,500 m·day^−1^ [3]. Because amino acids are also oxidized as energy sources at ~4.4% or 10% of the total exercise-induced energy expenditure in a carbohydrate-loaded or carbohydrate-depleted state, respectively [4], they have to be replaced by dietary protein and amino acid intakes. Moreover, some studies have indicated that competitive swimmers may not consume enough energy to meet their energy expenditure [3,5,6]. Since changes in protein metabolism result in increased protein requirements during energy deficit [7,8,9], competitive swimmers with higher training volumes may require higher protein intakes. In addition, since power correlates with swimming performance [10,11], participation in a combination of dry-land strength training as part of the training program is desirable for competitive swimmers in the improvement of muscle strength and power. In particular, a general preparation phase includes a large volume of resistance-type training to increase capacity and change body composition to perform exercise repetitions with a greater mechanical specificity [12]. A combination of interval training exercise and resistance exercise synergistically increases muscle protein synthesis (MPS) in female swimmers [13]. Since resistance training increases the MPS rate [14], which requires a protein substrate (i.e., amino acid intake after exercise), we hypothesized that the protein needs in athletes during the general preparation phase may be increased.

In general, greater protein intakes (1.2–2.0 g·kg^−1^·day^−1^) are recommended for athletes [15] compared to the current recommended daily allowance (RDA; 0.8 g·kg^−1^·day^−1^) [16], partly because of the associated increase in the amino acid oxidation rate during endurance exercise [17]. Generally, protein requirements are examined by the nitrogen balance (NBAL) technique [17]. In recent reports utilizing a novel technique, the indicator amino acid oxidation (IAAO) method indicated that the NBAL technique may underestimate true requirements [18]. However, since the IAAO method requires an isotopic steady state, it is not available for investigating protein metabolism in the free-living state. Thus, NBAL is still the gold standard method to assess protein requirements and protein metabolism in a free-living state. However, no study to date has investigated the NBAL during extremely high-volume swimming training combined with resistance exercise. Therefore, the primary aim of the current case study was to investigate the dietary nutritional intakes and NBAL in competitive collegiate swimmers in order to obtain practical information on the protein requirements and recommended protein intake.

Several recent sports science consensus statements have reported that the timing of protein or amino acid intake as well as the composition of the amino acids consumed could affect athletes’ protein metabolism [9,19,20]. According to the position stand of the International Society of Sports Nutrition [19], the ingestion of 20–40 g of protein (0.25–0.40 g·kg^−1^·dose^−1^) from a high-quality source or 10 g of free-form essential amino acids (EAAs) every 3 to 4 h appears to maximize the MPS rate for body composition and performance outcome improvements. Particularly, 700–3000 mg of leucine and/or a higher relative leucine content, with a balanced composition of EAAs should be consumed at each ingestion [9]. Therefore, it is important to assess the intake timing and composition of the amino acids consumed in meals and snacks during the day. Recently, by analyzing data from Japan’s 2012 National Health and Nutrition Survey, protein intake and daily distribution in Japanese adults aged over 30 years were investigated [21]. According to the report, more than 95% of the participants met the recommended protein levels, but protein and leucine intakes at breakfast were not sufficient [21]. However, no studies have investigated the amino acid compositions of meals and snacks in competitive athletes during training season.

Therefore, we investigated the daily nutrient intakes, with analyses of the intake timings and amino acid compositions of each meal and snack, as well as NBAL in competitive collegiate swimmers during the general preparation phase in order to generate practical knowledge regarding the protein requirements and recommendations for competitive swimmers.

## 2. Materials and Methods

### 2.1. Ethics Statement

All participants were informed of the purpose of the study, the experimental procedures involved, and all the potential risks involved before obtaining written consent. Similarly, participants aged under 19 years and their parents were also informed of the study procedures and any associated potential risks prior to their signing of written assent and consent documents, respectively. This study was conducted in accordance with the Declaration of Helsinki, and the protocol was approved by the research ethics board of the National Institute of Fitness and Sports and the institutional review board of Ajinomoto Co., Inc (No. 2017-014). Informed written consent was obtained from all participants aged over 20 years. Informed written assent was obtained from all participants aged under 20 years, and written informed consent was obtained from their parents. This trial was registered in UMIN Clinical Trials Registry [22].

### 2.2. Participants

Participants were recruited from among members of the swimming team of the National Institute of Fitness and Sports. Fourteen healthy trained male collegiate swimmers participated in the study. They trained routinely, participating in more than ten training sessions per week. However, one participant was excluded from the data analysis due to sample collection failure. The participants’ characteristics are described in Table 1. General anthropometric measurements (i.e., body weight, height, and body composition) were obtained during the five-day experimental period. Body weight and composition (i.e., fat mass and fat-free mass) were measured via bioelectrical impedance analysis (Omron HBF-701, OMRON HEALTHCARE Co., Ltd., Kyoto, Japan).

### 2.3. Experimental Design

**Figure 1:**
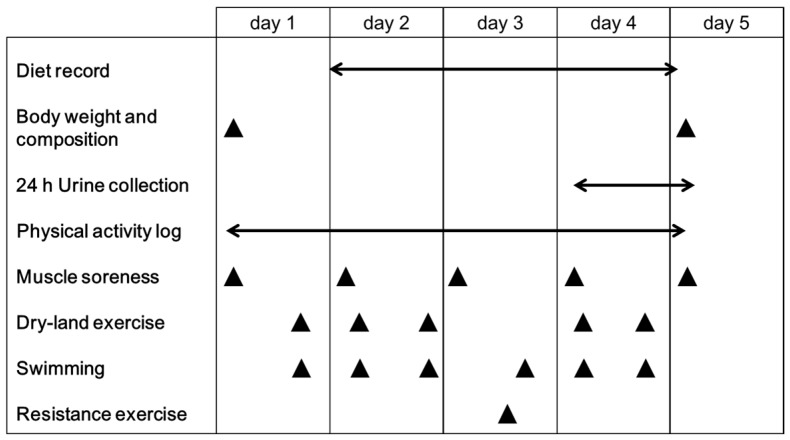
Schematics of the experimental protocol.

Each participant completed five days in the experimental period, and VO_2_max measurements and resting energy expenditure (REE) measurements were performed separately. This five-day experimental phase was conducted from 16 to 20 October 2017; the swimmers had resumed training at the start of October and had trained for two weeks by this time, and therefore had begun to regain their fitness levels after off season. The protocol involved in this phase is shown in Figure 1. Briefly, on the morning of the first and fifth days, participants arrived at the laboratory after an overnight fast and their body weight and body composition were measured. The participants performed training activities according to the training schedule provided by the swimming club coach and consumed their habitual diet ad libitum. The training schedule contains three types of exercise: dry-land exercise, swimming, and resistance exercise. Dry-land exercise aimed at improving muscle strength and included weight-bearing strength training. Morning exercise sessions were held from 5.30–7.30 a.m. on days 2 and 4, comprising 45 min of dry-land exercise and 75 min of swimming exercise. Evening sessions were held from 4.00–7.00 p.m. on days 1, 2, and 4, including 75 min of dry-land exercise and 105 min of swimming exercise. On day 3, resistance exercise (60–90 min) and swimming exercise (75 min) were performed. Resistance exercise comprised three sets of 8−12 reps of 80% one-repetition maximum (1RM) with 5-min rest intervals between sets for squat, bench press, deadlift, pulldown, dumbbell lateral raise, and leg press.

### 2.4. Energy Expenditure

The total daily energy expenditure was estimated by summing the REE, energy expenditure during normal daily physical activities (e.g., commuting, shopping, etc.), diet-induced thermogenesis (DIT), and exercise-induced energy expenditure (EEE) values. The REE value was obtained after the completion of the five-day experimental period. The participants’ resting oxygen consumption (VO_2_) and carbon dioxide production (VCO_2_) values were measured by the Douglas bag method. Fractions of O_2_ and CO_2_ in the expired gas were determined using an automated Vmax29c gas analyzer (SensorMedics Corporation, Yorba Linda, CA, USA). Expired gas volume was measured using a dry gas meter (Shinagawa Corporation, Tokyo, Japan). Calculations of REE were performed using VO_2_ and VCO_2_ values by the abbreviated Weir’s equation [23]. Participants were required to wear an accelerometer (wGT3X-BT, ActiGraph, Pensacola, FL, USA) all day (except when bathing and exercising) during the trial for the monitoring of their habitual physical activity. The DIT value was estimated from the total energy intake and protein-fat-carbohydrate (PFC) ratio in the diet according to the method followed in a previous study [24], since the PFC balance in the current study was similar to that observed in that report. A heart rate monitor (M430 or M600, Polar Electro, Kempele, Finland) was provided for participants to wear during dry-land and swimming exercise sessions to monitor the quantity and intensity of each exercise bout. EEE was calculated from the average heart rate (HR) and exercise duration, based on the REE and maximal energy expenditure values at VO_2_max testing. In particular, the EEE during resistance exercise was estimated based on Phillips’ study [25].

### 2.5. Dietary Intakes

Nutrient intake was assessed by the analysis of daily food records. Participants were instructed to maintain their usual eating habits over the experimental period. A registered dietician from our research team instructed participants on how to maintain thorough records. A detailed food record was designed to obtain qualitative and quantitative data on nutrient intakes over three days. Participants were also asked to record quantitative and qualitative data on all food and beverage intakes (including snacks) and the timings of consumption. All the participants returned the completed food records on the final day of the experimental period and then provided further details individually to the registered dietician. Participants described the average portion sizes of the foods, beverages, and supplements consumed. In addition, the main menu and a large portion of carbohydrate sources were weighed using a portable scale by the athletes themselves. Detailed descriptions of all the foods and beverages (including brand names) consumed, as well as their methods of preparation and cooking, were recorded. As the dietary intake on day 1 was not recorded, we applied the average dietary intakes over the next three days for day 1. Dietary intakes were assessed using Excel Eiyo-kun ver.8 (Kenpaku-sha, Tokyo, Japan), a software that contains the nutrient information of various foods or combination of selected foods and amino acid contents of some Japanese foods [26,27]. To analyze the intake quantity and timing of each amino acid over the day, the timing of consumption was categorized into seven eating occasions: pre-breakfast snack, breakfast, morning snack, lunch, afternoon snack, dinner, and pre-sleep snack.

### 2.6. Nitrogen Balance

On day 4, all the urine produced after the initial morning spot urine test up to the first urination the following morning was collected to determine NBAL. The collected urine was acidified by the addition of anhydrous citric acid into the collection bottle and stored at 4 °C until analysis. Creatine and urea concentrations were measured by Clinical Pathology Laboratory Inc. (Kanoya, Japan). The measured nitrogen excretion was the sum of the urinary urea nitrogen and creatinine nitrogen excretion, representing >75% of the total daily nitrogen excretion in athletes [28]. Miscellaneous nitrogen excretion (e.g., sweat, fecal) was estimated according to previously published values in a trained endurance running population consuming 1.7 g·kg^−1^·day^−1^ of protein [28]. NBAL was calculated as NBAL = E − I, where E represents nitrogen excretion and I represents total nitrogen intake, calculated as the protein intake on day 4 divided by 6.25—A factor used to convert grams of protein into grams of nitrogen.

### 2.7. Statistical Analysis

Values are reported as the mean ± standard deviation. Paired *t*-tests were used to determine the differences in body weight and body composition between days 1 and 5. To determine whether the energy balance (energy intake-total energy expenditure) was significantly positive or negative, a paired *t*-test was used to compare differences from zero for all the groups. Linear regression analyses were applied to NBAL data for the determination of the estimated average protein intakes required to achieve zero NBAL. Generally, recommended protein intake is set as the protein intake that is suitable for 97.5% of the population [29]. Thus, in an attempt to provide practical information applicable for competitive swimmers, the upper limit of the 95% confidence interval of the average protein intake was estimated using the coefficients of variation for maintenance and protein deposition. Repeated measures one-way analysis of variance following Tukey’s multiple comparisons test was used to determine differences in the dietary intake between each day. Data were analyzed using the GraphPad Prism 6 software (GraphPad Software Inc., San Diego, CA, USA); values of *p* < 0.05 were considered significant.

## 3. Results

### 3.1. Body Weight and Composition

Participants’ morphological data are shown in Table 2. While body weight did not change (*p* > 0.05), body composition (fat mass %) significantly changed during the five-day exercise protocol (*p* < 0.01). Fat mass significantly decreased and the FFM increased over this period (*p* < 0.01, 0.05, respectively).

### 3.2. Daily Dietary Intakes

Data on the dietary intakes on day 2, 3, and 4 and average intakes are shown in Table 3. The energy, protein, and carbohydrate intakes on day 2 (*p* < 0.01, 0.05, and 0.01, respectively) and day 4 (*p* < 0.01, 0.05, and 0.01, respectively) were higher than those on day 3. However, the ratio of the protein and carbohydrate intake to total energy intake was not significantly different across the days (*p* > 0.05). The fat intake on day 2 was higher than that on day 3 (*p* < 0.05).

**Figure 2:**
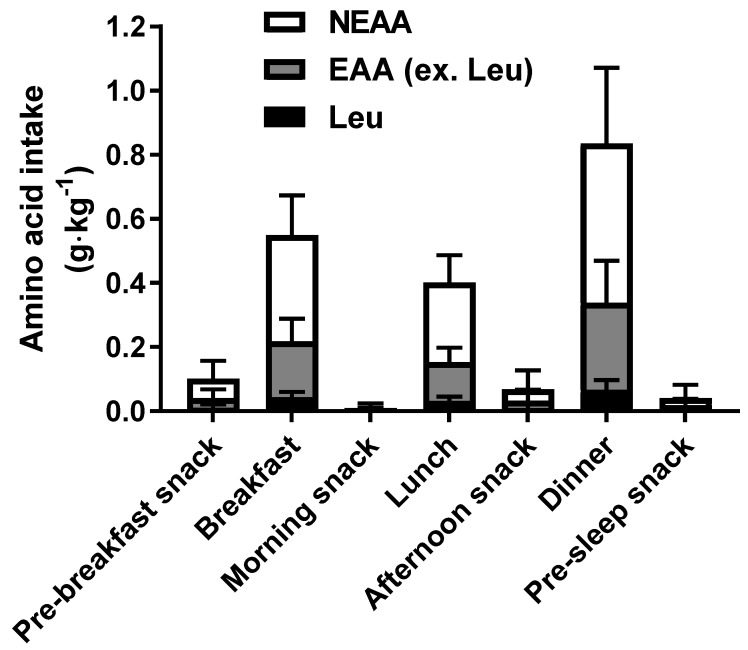
Distribution and composition of the dietary amino acid consumed throughout day 4. Data are shown as mean ± standard deviation (*n* = 13). EAA, essential amino acid; NEAA, non-essential amino acid; Leu, leucine.

The distribution of the composition of the dietary amino acids consumed throughout day 4 is shown in Figure 2. Dietary amino acids were divided into leucine alone, EAAs except for leucine, and non-essential amino acids. The protein intake amounts in the three main meals (breakfast, lunch, and dinner) were 0.57, 0.41, and 0.89 g·kg^−1^ (38.6, 27.9, and 59.8 g·meal^−1^), respectively. The leucine contents in the three meals were 0.044, 0.032, and 0.067 g·kg^−1^ (2.9, 2.2, and 4.5 g·meal^−1^), respectively, with essential amino acids other than leucine accounting for 0.18, 0.12, and 0.28 g·kg^−1^ (9.3, 8.7, and 23.8 g·meal^−1^), respectively. The protein intake amounts in the pre-breakfast, morning, afternoon, and pre-sleep snacks were 0.11, 0.01, 0.08, and 0.04 g·kg^−1^ (7.2, 0.7, 5.1, and 2.7 g·snack^−1^), respectively, with leucine accounting for 11, 0.7, 9.9, and 4.7 mg·kg^−1^ (731, 49.2, 681, and 318 mg·snack^−1^), respectively.

### 3.3. Energy Expenditure

The quantity and quality of the exercise sessions during the five days are summarized in Table 4. The total daily energy expenditure during the five-day experimental period was estimated to summarize the REE, DIT, and EEE (habitual physical activity and training sessions) values, as shown in Table 5.

### 3.4. Energy Balance

**Figure 3:**
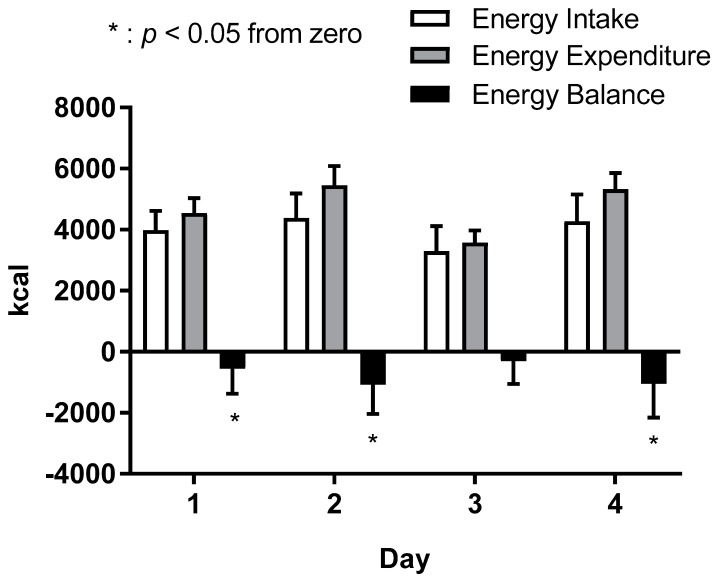
Energy balance (Energy intake − Energy expenditure) during the five-day experimental period. The energy balance was significantly negative on days 1, 3, and 4 (*p* < 0.05). Data are shown as mean ± standard deviation (*n* = 13). * *p* < 0.05 vs. zero.

The energy balance on each day was estimated by calculating the energy intake minus the total energy expenditure (Figure 3). Negative energy balance was observed on days 1, 2, and 4 (*p* < 0.05 for all).

### 3.5. Nitrogen Balance

**Figure 4:**
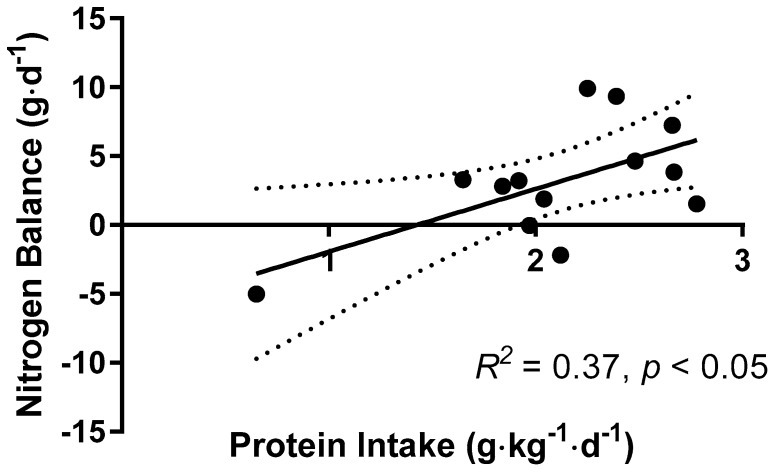
Relationship between nitrogen balance and protein intake.

The relationship between protein intake and NBAL is shown in Figure 4. A linear relationship was observed between the protein intake on day 4 and NBAL (*R*^2^ = 0.37, *p* < 0.05). The estimated average protein intake required for zero NBAL was calculated to be 1.43 g·kg^−1^·day^−1^, and the upper 95% confidence interval of the estimated protein intake, which estimates the population-safe protein intake, was estimated to be 1.92 g·kg^−1^·day^−1^.

The solid lines indicate the linear regression line of best fit, and the dashed lines represent the 95% confidence interval (CI). A significant positive correlation was observed (*R*^2^ =0.37, *p* < 0.05, *n* = 13). The estimated average protein intake for zero nitrogen balance was estimated to be 1.43 g·kg^−1^·day^−1^. The upper 95% CI of the estimated average protein intake, which estimates the population-safe protein intakes, was estimated to be 1.92 g·kg^−1^·day^−1^. The dashed lines show the 95% CI of the regression line.

## 4. Discussion

In the present study, we investigated the NBAL in competitive collegiate swimmers during the general preparation phase with analysis of protein and amino acid intake at each eating occasion, and estimated the average protein intake for zero NBAL and population-safe protein intake to be 1.4 and 1.9 g·kg^−1^·day^−1^, respectively. Recent reports indicated that the dose and timing of protein intake during the day could affect MPS. While the intake of 20 g of high-quality protein maximized the MPS after leg-based resistance exercise [31,32], the intake of 40 g of whey protein increased the MPS rate compared to the intake of 20 g of whey protein after whole-body resistance exercise [30]. Thus, in this current population that performed whole-body resistance exercise, 20–40 g of protein per meal were required. Furthermore, Areta et al. examined the differences in the MPS rates when participants ingested a total dose of 80 g of protein in different patterns over a 12-h measurement period following a bout of lower body resistance exercise [33]. The intake of 20 g of whey protein in four sittings induced higher MPS rates compared to the intakes of 10 g and 40 g of the same in eight and two sittings, respectively. As a result, 20–40 g (0.25–0.40 g·kg^−1^) of high-quality protein every three to four hours should be ingested after exercise [19]. Otherwise, using 2.2 g·kg^−1^·day^−1^ reported in the literature [34] spread out over the same four meals would necessitate a maximum of 0.55 g·kg^−1^·meal^−1^ [21]. Although the distribution pattern and composition of protein intake could affect protein requirements, no studies have investigated NBAL in athletes along with the distribution pattern and composition of protein intake. In this current study, the protein intake in the three main meals (breakfast, lunch, and dinner) in our current study met the level recommended in the guideline; however, the protein intake through snacks was lower than the recommended value. Particularly, since the protein intake at dinner was much higher than that (40 g) required for the maximization of MPS, the allocation of a part of the protein intake from dinner to the snacks consumed after training is recommended, as the gap between lunch and dinner was greater than 3–4 h. The allocation of protein intake may aid in the efficient utilization of proteins, decreasing the total daily protein needs. In addition, the quality of protein ingested has a significant effect. Animal-based proteins are considered to have greater anabolic properties than plant-based proteins [35,36], as the content of leucine—a potent stimulator of skeletal and liver protein synthesis through its ability to activate the mechanistic target of the rapamycin (mTOR) pathway [37,38]—is higher in animal-based protein [39]. To maximize MPS, 700–3000 mg of leucine should be consumed in each meal or snack. This is the first study to investigate the quality of protein (i.e., amino acid composition) among athletes and provides information on how to improve the diet to maximize MPS during the day. In the current study, while the leucine content in the three main meals and pre-breakfast snack were higher than 700 mg, the leucine content in the other snacks did not match the guideline. Thus, leucine supplementation during meals could optimize whole-day MPS in this current population. In literature, leucine supplementation induced higher gains in strength, but not in lean body mass, in novice trainees during 12 weeks of a weight training program [40]. Indeed, the ingestion of branched chain amino acids alone increased but did not maximally stimulate the MPS following exercise, as the lack of other EAAs limits the response of myofibrillar-MPS following exercise [41]. The intake of a leucine-enriched EAA mixture stimulates MPS compared to that of a conventional EAA mixture [42] and enhances recovery from exercise-induced muscle damage [43,44]. In addition, the enrichment of the branched-chain amino acid intake decreased the total amino acid dose required to meet the amino acid need in athletes undertaking endurance training during a three-day training protocol [45]. Thus, to supply other EAAs as substrates for protein synthesis, leucine or branched-chain amino acids should be co-ingested with other EAAs.

Current guidelines for athletes recommend the intake of 1.2–2.0 g·kg^−1^·day^−1^ of protein [9,15]. In the case of endurance athletes, the specific recommended protein intake was set as 1.2−1.4 g·kg^−1^·day^−1^ [17,46], largely based on the research examining the protein intakes required to achieve NBAL [28,47,48]. The population-safe protein intake in the current study was higher than that in previous studies [28,47]. There are several possible reasons for this difference. First, the swimmers participated in large volumes of training in the current study, possibly leading to increased protein requirements compared to those reported in previous studies. This increased requirement among athletes on endurance training reflects the need to replace the oxidative amino acid loss during exercise [17] and provide substrates for the increased synthesis of muscle protein and other body proteins after exercise [49]. While the participants of previous studies trained for approximately 1–2 h·day^−1^ [27,46,47], our participants trained for 6 h/day. Although the exercise modalities varied across the studies, the energy expenditure on the experimental day in the current study (5500 kcal·day^−1^) was significantly higher than that in previous studies (4200–4500 kcal·day^−1^) [28,47]. Thus, according to the increased oxidative amino acid loss during exercise, the protein needs may be higher in the current study than in previous studies [28,47]. Second, in the current study, the participants had negative energy balance. Energy deficit results in an increase in the rates of whole-body proteolysis, amino acid oxidation, nitrogen excretion [50,51], and the suppression of MPS [52,53,54]. Changes in protein metabolism may result in increased protein requirements during energy deficit [7,8,9]. In addition, our participants had lower carbohydrate intakes than recommended during the high-volume endurance training phase [15]. Low carbohydrate availability potentially increases the contribution of endogenous protein to energy provision [4,55] and suppresses MPS [55]. These factors may have led to the increased protein requirements in our study. Third, the modality of exercise could affect the protein requirement. During the general preparation phase, the participants executed large volumes of dry-land strength-type exercises in order to increase their free-fat mass in preparation for the specific preparation phase. According to the previous position stand by the American College of Sports Medicine, the protein requirements of athletes undertaking resistance training is higher than that for athletes undertaking endurance training [16]. Therefore, participation in a large volume of strength-type training may have induced the increased protein requirement in our swimmers. We recruited only competitive collegiate swimmers in the current study. Their VO_2_max values were similar to those observed in a previous study [2] and the training volume and daily energy expenditure were similar to those in elite world-class swimmers [3]. Thus, the knowledge obtained in this study can be applicable to competitive swimmers. However, the energy expenditure during swimming varies depending on factors such as swimming skill [56]. Thus, future studies should focus on the protein requirements in novice swimmers or top elite swimmers.

Protein requirements are classically estimated based on nitrogen balance studies, with at least two levels of protein intake for several days up to 2 weeks [28,57,45]. In former studies, Boisseau et al. reported that protein intake for achieving a zero NBAL (as determined by dietary analysis and urinary excretion, similar to the current study) was lower than the protein requirements determined by nutritional intervention studies [45,58]. In our current study, we investigated NBAL in competitive swimmers in the free-living state to understand habitual protein intake and metabolism. We found that a protein intake for achieving zero NBAL was 1.4 g·kg^−1^·day^−1^ in this population based on dietary analysis and urinary nitrogen excretion. Therefore, further studies that look into a diet covering less than and more than 1.4 g·kg^−1^·day^−1^ protein are warranted in order to determine protein requirements in this population. Furthermore, protein requirements are classically determined by the NBAL technique, which determines the protein intake required for the achievement of zero NBAL. However, this method may have limitations [59], including a general predisposition to overestimate nitrogen intake and underestimate nitrogen excretion [60], which may collectively result in an underestimation of the true protein requirement [18]. The IAAO method was developed as an alternative to the NBAL technique to assess the individual amino acid and protein recommendations in a variety of populations [61,62]. Recently, the IAAO method was applied in athletic populations to determine protein and amino acid requirements [63,64,65,66]. The IAAO method determined the protein requirements and recommended protein intakes for strength athletes at 1.7 and 2.2 g·kg^−1^·day^−1^ [64] and for endurance athletes as 1.6 and 1.8 g·kg^−1^·day^−1^, respectively [65]. The recommended protein intake determined by the recommended protein intake was 30%–50% greater than previous recommended levels for populations on endurance training based on NBAL data [16,65]. The IAAO method defines protein requirement as the protein intake required for the maximization of whole-body protein synthesis, which could be consistent with the protein intake for the maximization of the NBAL [18]. In addition, a recent report indicated that the recommended protein intake determined by the IAAO method may be appropriate to improve the exercise performance in athletes on endurance training compared to the protein intake required for zero protein balance [67]. According to the concept of optimal protein intake determined by the IAAO method, the protein intake for the performance optimization in this study’s competitive swimmers may be 2.4–2.8 g·kg^−1^·day^−1^, estimated based on the recommended protein intake determined by the NBAL, multiplied by 130%–150%. Further study is needed to validate the optimal protein intake required for the optimization of the exercise performance in such populations.

## 5. Conclusions

In conclusion, during the general preparation phase, which comprises a large volume of dry-land strength exercise, the protein requirement in competitive swimmers for the achievement of zero NBAL is 1.4 g·kg^−1^·day^−1^ and the population-safe protein intake was found to be 1.9 g·kg^−1^·day^−1^, which is in line with the current recommended protein intake [14,23]. Since the daily intake patterns of protein and amino acids were not ideal, the allocation of proteins and amino acids from dinner to the snacks consumed between lunch and dinner, or the intake of amino acids supplements, especially leucine-enriched supplements in the afternoon snack, may decrease the daily protein requirement. Further study is needed to validate the optimal protein and amino acid intakes required within the day for the optimization of exercise performance.

## Figures and Tables

**Table 1:** Initial characteristics of the participants.

	Mean ± SD (*n* = 13)
Age (year)	19.7 ± 1.0
Height (cm)	172.8 ± 5.1
Body weight (kg)	67.4 ± 5.1
FFM (kg)	57.0 ± 3.5
Body Fat (%)	15.4 ± 3.3
VO_2_max (mL·kg^−1^·min^−1^)	63.9 ± 3.7

Data are shown as mean ± SD. SD, standard deviation; VO_2_max, maximal oxygen consumption; FFM, fat-free mass.

**Table 2:** Body weight and body composition changes.

	Day 1	Day 5	*p*-Value
Body weight, kg	67.4 ± 5.1	67.7 ± 5.2	>0.05
Fat mass, %	15.4 ± 3.3	14.1 ± 2.4	<0.01
Fat mass, kg	10.5 ± 2.9	9.6 ± 2.3	<0.01
Fat-free mass, kg	57.0 ± 3.5	58.0 ± 3.6	<0.05

Data are shown as mean ± standard deviation (*n* = 13).

**Table 3:** Average daily energy intake and macronutrient breakdown.

	Day 2	Day 3	Day 4 (Test Day)	Average
Energy				
kcal·day^−1^	4385 ± 803 **	3279 ± 817	4279 ± 876 **	3981 ± 629
Protein				
g·day^−1^	141.7 ± 27.7 *	105.8 ± 37.5	142 ± 39.3 *	129.8 ± 25.9
g·kg^−1^·day^−1^	2.1 ± 0.4 *	1.6 ± 0.6	2.1 ± 0.6 *	1.9 ± 0.4
Energy %	13 ± 2	13 ± 2	13 ± 2	13 ± 1.4
Fat				
g·day^−1^	153.2 ± 32.5 *	110.8 ± 47.6	155.9 ± 47.7	140 ± 30
g·kg^−1^·day^−1^	2.3 ± 0.6 *	1.7 ± 0.8	2.4 ± 0.8	2.1 ± 0.5
Energy%	33 ± 4	31 ± 6	35 ± 6	31.5 ± 3.8
Carbohydrate				
g·day^−1^	589.1 ± 123 **	449.3 ± 84.4	553.4 ± 111.1 **	530.6 ± 86.8
g·kg^−1^·day^−1^	8.7 ± 1.8 **	6.7 ± 1.3	8.2 ± 1.7 **	7.9 ± 1.3
Energy %	54 ± 5	56 ± 7	52 ± 6	53 ± 4

Data are shown as mean ± standard deviation (*n* = 13). *, **: significant difference compared to day 3, *p* < 0.05 and 0.01, respectively.

**Table 4:** Summary of exercise sessions during the five-day experimental period.

		Morning Sessions		Evening Sessions	
		Dry-Land	Swimming	Dry-Land	Swimming
day 1	HR (bpm)			112 ± 15	134 ± 6
	Time (mins)			82 ± 5	117 ± 3
	Distance (m)				5500
day 2	HR (bpm)	92 ± 8	124 ± 5	101 ± 14	129 ± 7
	Time (mins)	55 ± 8	90 ± 6	76 ± 10	122 ± 1
	Distance (m)		4400		5500
day 3	HR (bpm)				119 ± 7
	Time (mins)				57 ± 9
	Distance (m)				2000
day 4	HR (bpm)	91 ± 8	118 ± 7	99 ± 11	127 ± 7
	Time (mins)	55 ± 6	92 ± 5	80 ± 11	121 ± 2
	Distance (m)		4400		5500

Data are shown as mean ± standard deviation (*n* = 13). HR, average heart rate (bpm) during exercise session; Time, duration (minutes) of the exercise session; Distance, distance (m) participants covered during the swimming exercise session.

**Table 5:** Energy expenditure during the five-day experimental period.

Kcal/day	Day 1	Day 2	Day 3	Day 4
Resting energy expenditure	1975 ± 245			
Diet-induced thermogenesis	283 ± 45 *	312 ± 57	233 ± 58	304 ± 63
Exercise-induced energy expenditure				
Habitual physical activity	446 ± 181	481 ± 215	481 ± 215	456 ± 141
Exercise sessions	1838 ± 283	2691 ± 371	926 ± 98	2589 ± 355
Total energy expenditure	4541 ± 498	5457 ± 629	3579 ± 401	5323 ± 529

Data are shown as mean ± standard deviation (*n* = 13). Diet-induced thermogenesis was calculated from the total energy intake times 7.1% [30]. * The average energy intake over days 2 to 4 was applied for day 1.
